# Combined effect of endophytic *Bacillus mycoides* and rock phosphate on the amelioration of heavy metal stress in wheat plants

**DOI:** 10.1186/s12870-024-04812-3

**Published:** 2024-02-20

**Authors:** Asim Shahzad, Uzma Aslam, Shazia Ferdous, Mingzhou Qin, Anam Siddique, Motsim Billah, Muhammad Naeem, Zahid Mahmood, Sadaf Kayani

**Affiliations:** 1 The College of Geography and Environmental Sciences, Henan University, Jinming ave, Kaifeng, China; 2 Department of Botany, Mohi-Ud-Din Islamic University, Nerian Sharif, Azad Jammu and Kashmir Pakistan; 3 Directorate of ORIC, Rawalpindi Women University, Rawalpindi, Pakistan; 4 Department of Plant Science, School of Agriculture and Biology, Shanghai Jiao Tong University, 800 Dongchuan Road, Shanghai, China; 5 Crop Sciences institute, National Agricultural Research Centre, Islamabad, Pakistan

**Keywords:** Heavy metals, Toxicity, Superoxide dismutase, Peroxidase, *Bacillus mycoides*, R*P*

## Abstract

**Background:**

Zinc (Zn) and nickel (Ni) are nutrients that are crucial for plant growth; however, when they are present at higher concentrations, they can cause toxicity in plants. The present study aimed to isolate plant growth promoting endophytic bacteria from *Viburnum grandiflorum* and assess its plant and defense promoting potential alone and in combination with R*P* in zinc (Zn) and nickel (Ni) toxic soil. The isolated endophytic bacteria were identified using 16s rRNA gene sequencing. For the experiment, twelve different treatments were applied using Zn, Ni, isolated endophytic *Bacillus mycoides* (Accession # MW979613), and rock phosphate (RP). The Ni, Zn and R*P* were used at the rate of (100 mg/kg) and (0.2 g/kg) respectively. A pot experiment with three replicates of each treatment was conducted using a complete randomized design (CRD).

**Results:**

The results indicated that Ni (T5 = seed + 100 mg/kg Ni and T9 = seed + 100 mg/kg Zn) and Zn concentrations inhibited plant growth, but the intensity of growth inhibition was higher in Ni-contaminated soil. *Bacillus mycoides* and R*P* at 100 mg/Kg Zn (T12 = inoculated seed + 100 mg/kg Zn + R*P*0.2 g/kg.) increased the shoot length, leaf width, protein and sugar content by 57%, 13%, 20% and 34%, respectively, compared to the control. The antioxidant enzymes superoxide dismutases (SOD), peroxidase (POD) were decreased in contaminated soil. Furthermore, Ni and Zn accumulation was inhibited in T11 (seed + 100 mg/kg Zn + R*P*0.2 g/Kg) and T12 (inoculated seed + 100 mg/kg Zn + R*P*0.2 g/Kg) by 62 and 63% respectively. The Cu, Ca, and K, contents increased by 128, 219 and 85, Mn, Na, and K by 326, 449, and 84% in (T3 = inoculated seed) and (T4 = inoculated seed + R*P* 0.2 g/Kg) respectively.

**Conclusions:**

Ni was more toxic to plants than Zn, but endophytic *bacteria* isolated from *Viburnum grandiflorum*, helped wheat (*Triticum aestivum*) plants and reduced the toxic effects of Ni and Zn. The effect of *Bacillus mycoides* was more prominent in combination with R*P* which promoted and suppressed heavy-metal toxicity. The reported combination of *Bacillus mycoides* and R*P* may be useful for improving plant growth and overcoming metal stress.

**Supplementary Information:**

The online version contains supplementary material available at 10.1186/s12870-024-04812-3.

## Introduction

Industrial emissions comprise toxic heavy metals in large amount, which are adequate to impact noxious effects on crop plants [1]. Metals such as copper, zinc, manganese, iron, and boron are micronutrients that are necessary for plant life and growth. These bio-elements are sometimes toxic or poisonous to plants health when they are present at higher concentrations [2]. Toxic heavy metals are key environmental adulterants, and their toxins are of increasing concern for environmental, evolutionary, and nutritional purposes [3, 4]. Heavy metals also have serious impacts on soil biology by the interface between the infectious procedure and soil microorganisms [5, 6].

Beneficial soil microorganisms in cultivated lands, invertebrates, and mammals are affected by metal toxicity [7, 8]. Higher concentrations of heavy metals such as Zn and Ni in soil [9] have a negative impact on soil micro-nutrients, and as a result of this stress condition, plant enzyme activity [10] stagnates plant growth which causes leaf necrosis in the older plants, degrades plant biomass, inhibits plant cell elongation and cell division [11, 12]. Bacteria that populate the interior parts of plants either mutualistically or symbiotically are called endophytic bacteria [13, 14]. There is a massive population of microorganisms found near the plant root zone, and these endophytes that enter plants from the soil must be efficient root invaders [15]. Endophytic Bacteria are located in different parts of plants within the cells or spaces among the cells, and are found in either below or above-ground parts of plants [16].

Phosphorus (P) is the second largest dietary supplement for crop plants and plays a significant biological role in plant respiration, cell division, photosynthesis, cell enlargement, synthesis of nucleic acids, synthesis of proteins, and adenosine triphosphate (ATP) in different plant species [17]. During the initial time of plants seedling development, seed phytate phosphorus is hydrolyzed, and non-phytate phosphorous is then remobilized to aid crop plant seedlings [18]. Poor phosphorous absorption in plants exposed definitely not distinctions in seedling vigor, plant mass, and profit. High absorption of phosphorous in plant seeds indicates that the genotypes are sensitive [19]. Rock Phosphate (R*P*) is an imperative biological origin of phosphorus which operates as a natural constituent for the generation of chemical phosphate manures [20]. R*P* is acceptable for acid-loving soils, as with low pH, it works in dissolving the R*P* and gaining the quantity available to plants [21]. Some bacterial strains that are solubilized to phosphate are capable of plant growth stimulating traits and enhance the plant biomass up to 20–40% [22]. The combined application of R*P* and inoculation of microbes is an emerging combination and the best technology to use organic R*P* for plant promotion as well as for a healthier atmosphere [23]. Rhizospheric and endophytic bacteria, of the *Pseudomonas, Bacillus*, and *Rhizobium species* are among the most influential phosphate-dissolving bacteria [24]. For both plants and microorganisms, the two basic characteristics attributed to the solubility of R*P* are the secretion of H ^+^ and generation of organic acids. Plant growth-promoting rhizobacteria (PGPR) have positive effects on plant growth and metabolism which can enhance crop productivity [25]. The induction of beneficial bacteria in plants has been known for many years; however, only technological advances that have occurred in the last decade have allowed researchers to understand and explore the full potential of these microorganisms [26] *Bacillus* spp. is known for their wide range of characteristics which represent about 75% of bio-pesticides manufactured worldwide [27] However, *Bacillus* spp have an underexplored potential to promote plant growth, as recently reported by some researchers [28]. Recent studies demonstrated that the bacterial production of indole-3-acetic acid (IAA) plays an important role in promoting plant growth. Bacterial IAA may influence plant growth by affecting processes such as cell division, elongation, tropism, apical dominance, senescence, flowering, and response to stress. IAA synthesized by beneficial bacteria, including *Bacillus* strains, may originate from five tryptophan-dependent metabolic pathways: indole-3-acetamide (IAM), indole-3-acetonitrile (IAN), tryptophan side chain oxidase (TSO), tryptamine (TAM), and indole-3-pyruvate (IPyA) [29].

Wheat (*Triticum aestivum* L.) is a hexaploid and is the third most popular staple food crop in the world after maize (*Zea mays*) and rice (*Oryza sativa*). Wheat is an important contributor to global energy and micronutrient intake. Consumption of wheat grain is more notorious than wheat flour because wheat loses valuable nutrients during the grinding process. Globally, 79% of the total wheat production comes from China, America, Turkey, Canada, Australia, India, and Argentina [30]. The production and quality of wheat plants depend on their genotype, environmental factors, and interactions between and abiotic stress is classified as an environmental factor that affects wheat yield. As a result of stress factors, the growth of wheat plants may be disturbed, causing stunted growth in wheat plants [31]. Plants are ironic in nature because of their anti-oxidant enzymatic activity. Antioxidant enzymatic defense activity is differing from plant to plant [32]. Anti-oxidants enzymes are proficient in preventing radical reactions and inhibiting changes in cell structure, and, in slight meditation, then substrates that are oxidized, significantly [33]. Different molecules react with reactive oxygen species (ROS), but elimination productivity is complex in enzymatic reactions, mainly catalyzed by ascorbate oxidase, superoxide dismutase (SOD), and catalase etc. ( [34]. Antioxidant enzymes at the molecular level directly and indirectly eradicate ROS. Specifically, chelation of transition metals is indirect mechanisms Haber-Weiss [35] while donating or accepting of electrons, foraging radicals, and stopping to react with biotic molecules are direct mechanism. In many circumstances, the total volume of these enzymes remains significantly lowered within some organelles in oxidized forms amassing in partitions with a less successful redox recycling process [34] Similar to other plants, wheat plants are also sensitive to metal stress. Once wheat interacts with heavy metals it triggers stress and affects plant growth, germination, plant biochemicals and eventually the loss of crop yield to biochemical responses and yield losses in wheat. Various heavy metals such as Ni and Zn have been found to be highly dangerous to wheat, resulting in growth inhibition and production of reactive oxygen species (ROS). Accumulation of ROS in wheat tissues, results in cell death [36].

Many crops have the ability to grow better and produce greater yields under low soil Zn conditions than others. Plants with sensitivity to yield on soil Zn supply are defined as Zn-efficient plant species [37]. Higher concentrations of Zn influx rates for a Zn-efficient genotype than for an inefficient genotype under low Zn conditions in some plants [37]. Since the nickel (Ni) is chemically similar to Zn is supposed to compete for similar binding sites with low selectivity, Ni-induced Zn deficiency may occur where high soil Ni concentrations inhibit Zn uptake through low-affinity uptake systems. Dalir et al. [38] suggested that Ni and Zn partially use the same carriers for uptake in soybeans. In a more recent study, relatively low concentrations caused very strong competition between Ni and Zn for uptake sites. Endophytic Bacterial strains isolated from different medicinal plants found to be impressive in enhancing plant growth, can improve the growth by producing plant hormones, making nutrients available, suppressing harmful pathogens and inducing stress tolerance against biotic and biotic factors. Under the natural condition, plants face different types of biotic and biotic stresses. Aswini et al. [39] reported that when wheat seeds were inoculated with different endophytic strains, wheat growth and seed grain growth increased under low metal stress conditions. Therefore, the responses at higher concentrations should be investigated. Studies on the use of raw phosphate and endophytic bacteria for the amelioration of heavy metals in plants are limited. The use of raw phosphate along with new endophytic plant growth-promoting bacteria is a novel approach to mitigate the toxic effects of heavy metals at the early seedling stages of the plant. Keeping in mind the importance of beneficial endophytic bacteria and raw phosphate usage for the enhancement of plant growth, the present study was designed to isolate the endophytic bacteria from the medicinal plant *Viburnum grandiflorum* roots and investigate the isolated endophytic bacteria for its plant growth-promoting and heavy metal detoxification potential in combination with R*P* in heavy metal contaminated soil.

## Results

### Identification and 16s rRNA sequencing of endophytic bacteria

Endophytic bacterial colonies isolated from *Viburnum grandiflorum* were subjected to 16 S rRNA sequencing. The colonies showed a 90% similarity with *Bacillus mycoides.*

### Phylogenetic relation of the isolated bacteria from *Viburnum grandiflorum*

**Fig. 1:**
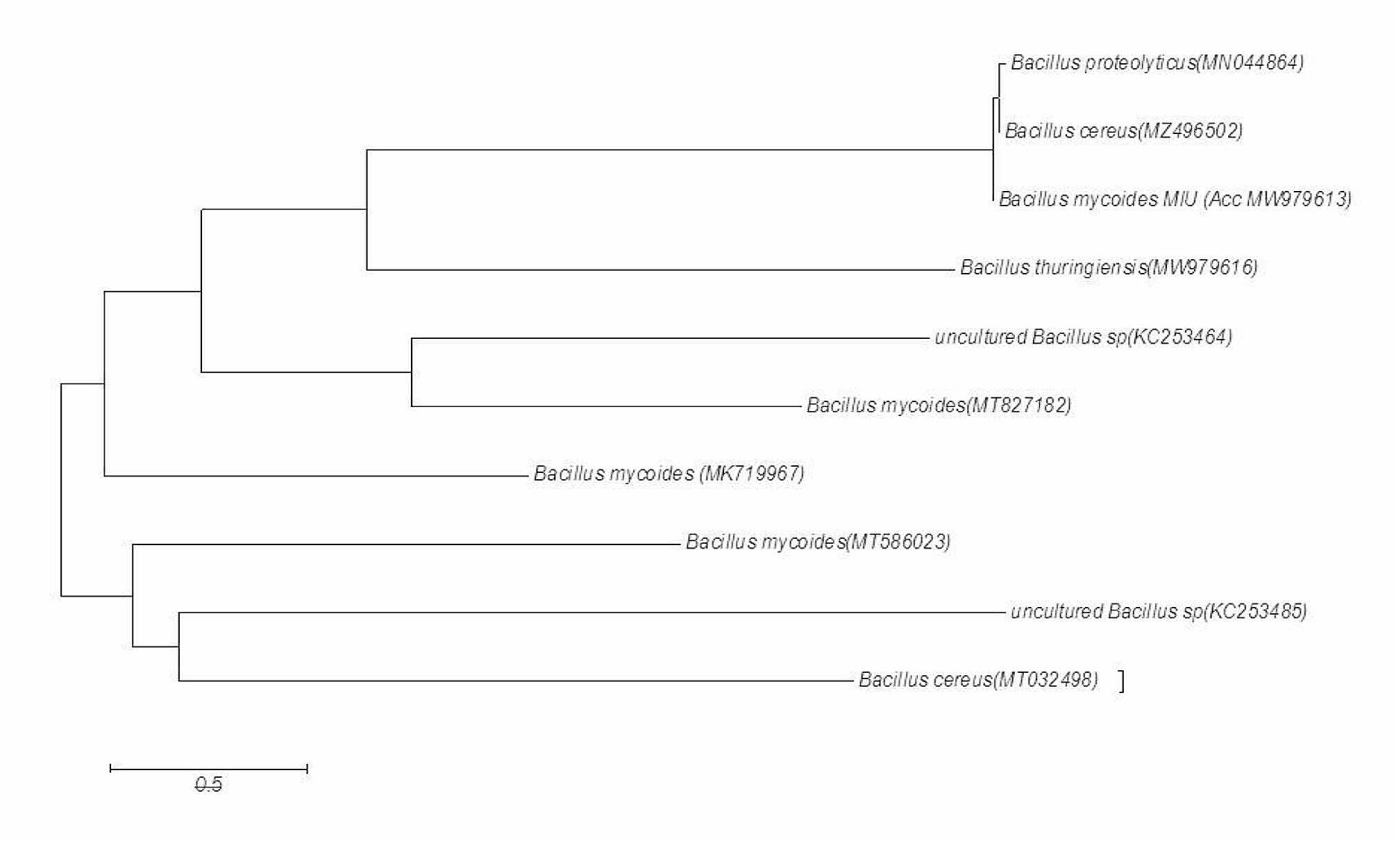
Neighbor-joining tree of *Bacillus mycoides* Isolated from Viburnum grandiflorum

*Bacillus mycoides* isolated from *Viburnum grandiflorum* were closely associated with agricultural soil bacteria (AJ252571) from (Germany). There were 10 positions in the final dataset. In the second clade, one species from India is closely related to that from Korea. In the next clade, the two species from China were closely related. These two species are related to Korean species. In the next clade, one species from India was closely related to that of France. *Bacillus subtilis* branched off from a group of clades during evolution (Fig. 1).

### Effects of RP and bacterial inoculation on germination percentage, shoot length, root length, and number of leaves under Zn and Ni stress

**Fig. 2:**
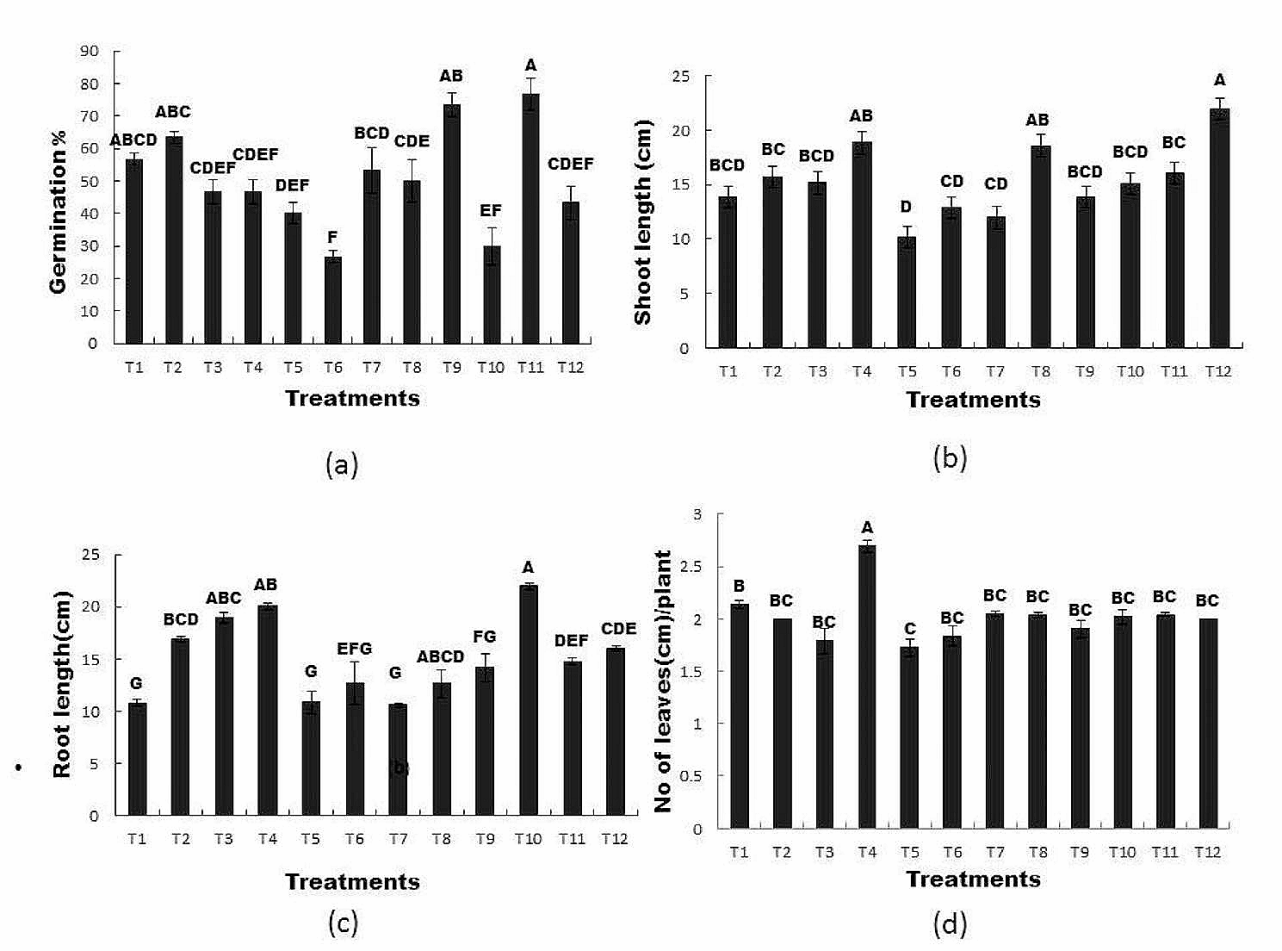
The Effect of R*P* and bacterial inoculation on (**a**) Germination percentage (**b**) shoot length (**c**) root length and (**d**) No of leaves under Zn and Ni stresss

The germination percentage of wheat seeds varied between the inoculated and uninoculated seeds. The addition of Ni and Zn inhibited seed germination. However, inoculation of seeds with *Bacillus mycoides* and application of R*P* enhanced the germination percentage in the presence of Ni and Zn. The germination percentage in T11 (seed + 100 mg Zn + R*P* 0.2 g/Kg) and T12 (inoculated seed + Zn + R*P*) was 35% and 57% higher than that in the control (T1) respectively (Fig. 2a). Shoot length in treatments inoculated with *Bacillus mycoides* and the application of R*P* significantly affected shoot length compared to uninoculated seeds. The shoot length was inhibited in the presence of Ni and Zn (T5 and T9); however, the inoculation of seeds with *Bacillus mycoides* and R*P* significantly increased the shoot length (T12, T8, T4), and % increase was 57%, 44%, and 36% higher than control, respectively (Fig. 2b). Root length was also affected by the different treatments. Inoculation with *Bacillus mycoides* and R*P* significantly affected root length compared to uninoculated seeds. The combined application of *Bacillus mycoides* and R*P* in T4 (inoculated seed + R*P* 0.2 g/Kg) significantly increase the root length of wheat but the addition of Zn and Ni inhibited the root length (T5, T7), However, the application of R*P* and inoculation of *Bacillus mycoides* reduced the effect of Zn and the maximum root length was observed in T10 (inoculated seed + Zn100 mg/Kg) which showed 102% and 55% increase in root length as compared to control and T9 respectively (Fig. 2c). The number of leaves was affected by inoculation with *Bacillus mycoides* and the application of R*P* compared to uninoculated seeds. The number of leaves decreased in the presence of Ni and Zn (T5); however, inoculation of seeds with *Bacillus mycoides* and application of R*P* significantly increased the number of leaves in T4 (inoculated seed + RP 0.2 g/Kg), which was 56% higher than that of T5. Similarly, in treatment T5 (uninoculated seed + Ni100 mg/kg), Ni significantly affected the number of plant leaves, which was 19% lower than that in the control. The results in Fig. 2 indicate that the combined application of *Bacillus mycoides* alone and in combination with R*P* significantly improved the germination percentage, shoot length, root length, and number of leaves per plant in Ni- and Zn-contaminated soils.

All treatments sharing common letters with similar bar patterns are similar; otherwise, they vary significantly (*P* < 0.05).

T1 = control, T2 = seed + R*P* 0.2 g/Kg, T3 = inoculated seed, T4 = inoculated seed + R*P* 0.2 g/Kg, T5 = seed + 100 mg/Kg Ni, T6 = inoculated seed + 100 mg/Kg Ni, T7 = seed + Ni100mg/Kg + R*P*0.2 g/Kg, T8 = inoculated seed + 100 mg/Kg Ni + R*P*0.2 g/Kg, T9 = seed + 100 mg/Kg Zn, T10 = inoculated seed + 100 mg/Kg Zn, T11 = seed + 100 mg/Kg Zn + R*P*0.2 g/Kg, T12 = inoculated seed + 100 mg/Kg Zn + R*P* 0.2 g/Kg.

### Effect of RP and bacterial inoculation on (a) leaf width, (b) plant height, and (c) leaf fresh weight under Zn and Ni stress

**Fig. 3:**
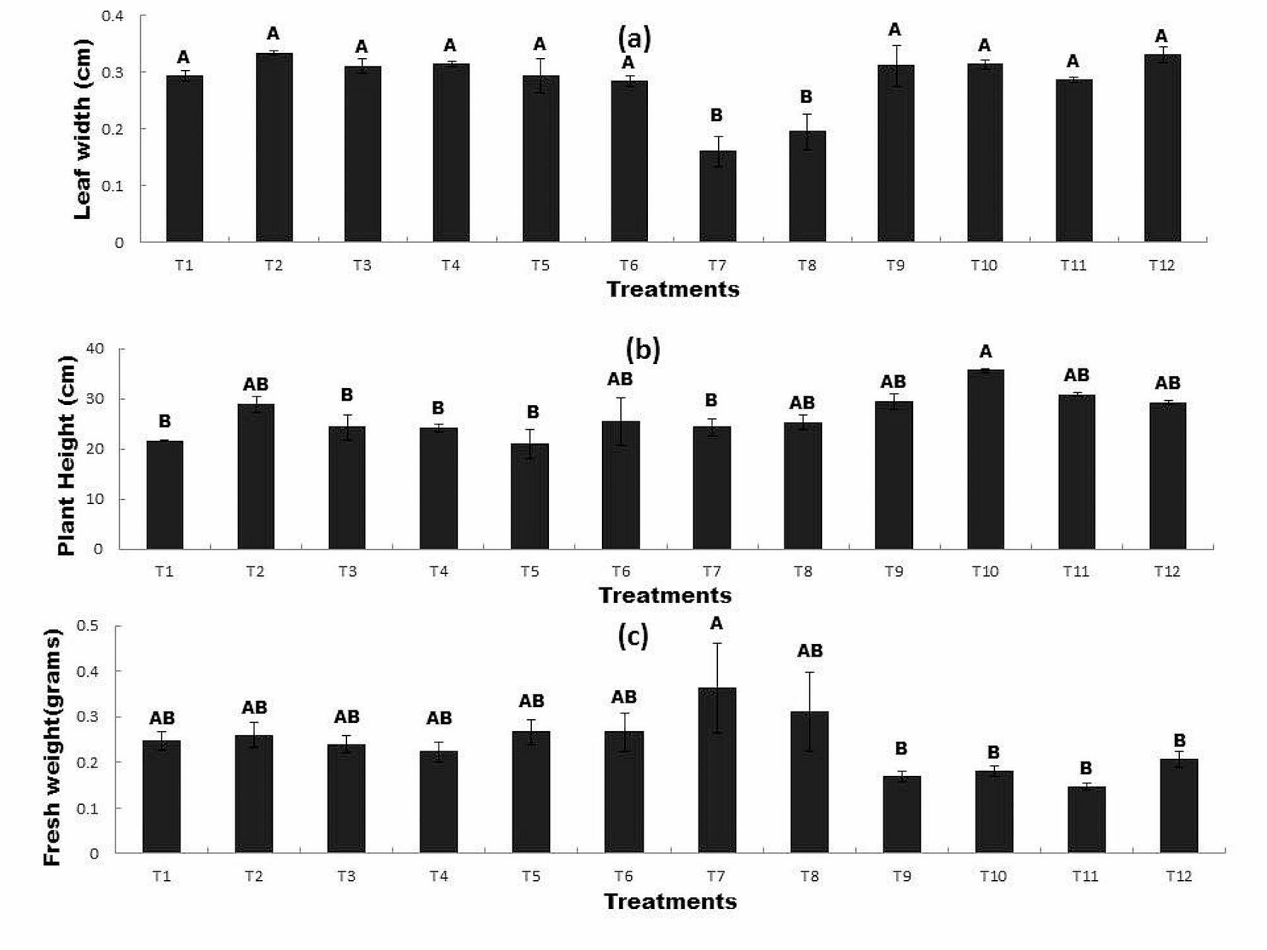
The Effect of RP and bacterial inoculation on (**a**) leaf width (**b**) plant height and (**c**) leaf fresh weight under Zn and Ni stress

Inoculation with *Bacillus mycoides* the application of R*P* significantly affected leaf width compared to uninoculated seeds. The addition of Ni significantly inhibited leaf width (T7). The maximum leaf width was observed in T2 (uninoculated seed + R*P* 0.2 g/Kg), which showed a 14% increase compared to that of control. Similarly, the combined application of *Bacillus mycoides* and R*P* in T4 (inoculated seed + Zn100mg/Kg + R*P* 0.2 g/Kg) also significantly increased the leaf width by 13% as compared to the control (Fig. 3a). The plant height of wheat varied between inoculated and uninoculated seeds. The addition of Ni decreased plant height (T5). However, the inoculation of seeds with *Bacillus mycoides* and the application of R*P* enhanced (T2, T6) plant height in the presence of Ni and Zn. The combined application of *Bacillus mycoides* and R*P* also inhibited plant height (T12). The maximum plant height was observed in T10 (inoculated seed + Zn100mg/Kg) followed by T11 (uninoculated seed + Zn100mg/Kg + R*P* 0.2 g/Kg) and the increase was 65% and 43% higher respectively than that of the control (Fig. 3b). Inoculation with *Bacillus mycoides* and application of R*P* significantly affected the leaf fresh weight compared to uninoculated seeds. The leaf fresh weight was inhibited in the presence of Ni and Zn (T11). However, the inoculation of seeds with *Bacillus mycoides* and application of R*P* significantly increased the leaf fresh weight (T7 and T8) and showed 47% and 26% higher leaf fresh weight respectively as compared to the control (Fig. 3c). Figure 3 indicates that leaf width and plant height were higher in T10 whereas, the fresh weight was found to be maximum in T7.

All treatments sharing common letter with similar bar pattern are similar otherwise vary significantly at *p* < 0.05.

T1 = control, T2 = seed + R*P* 0.2 g/Kg, T3 = inoculated seed, T4 = inoculated seed + R*P* 0.2 g/kg, T5 = seed + 100 mg/Kg Ni, T6 = inoculated seed + 100 mg/Kg Ni, T7 = seed + Ni100mg/Kg + R*P*0.2 g/Kg, T8 = inoculated seed + 100 mg/kg Ni + R*P*0.2 g/Kg, T9 = seed + 100 mg/Kg Zn, T10 = inoculated seed + 100 mg/Kg Zn, T11 = seed + 100 mg/Kg Zn + R*P*0.2 g/Kg, T12 = inoculated seed + 100 mg/Kg Zn + R*P*0.2 g/Kg.

### The Effect of R*P* and bacterial inoculation on (a) protein (b) sugar and (c) proline content of plant under Zn and Ni stress

**Fig. 4:**
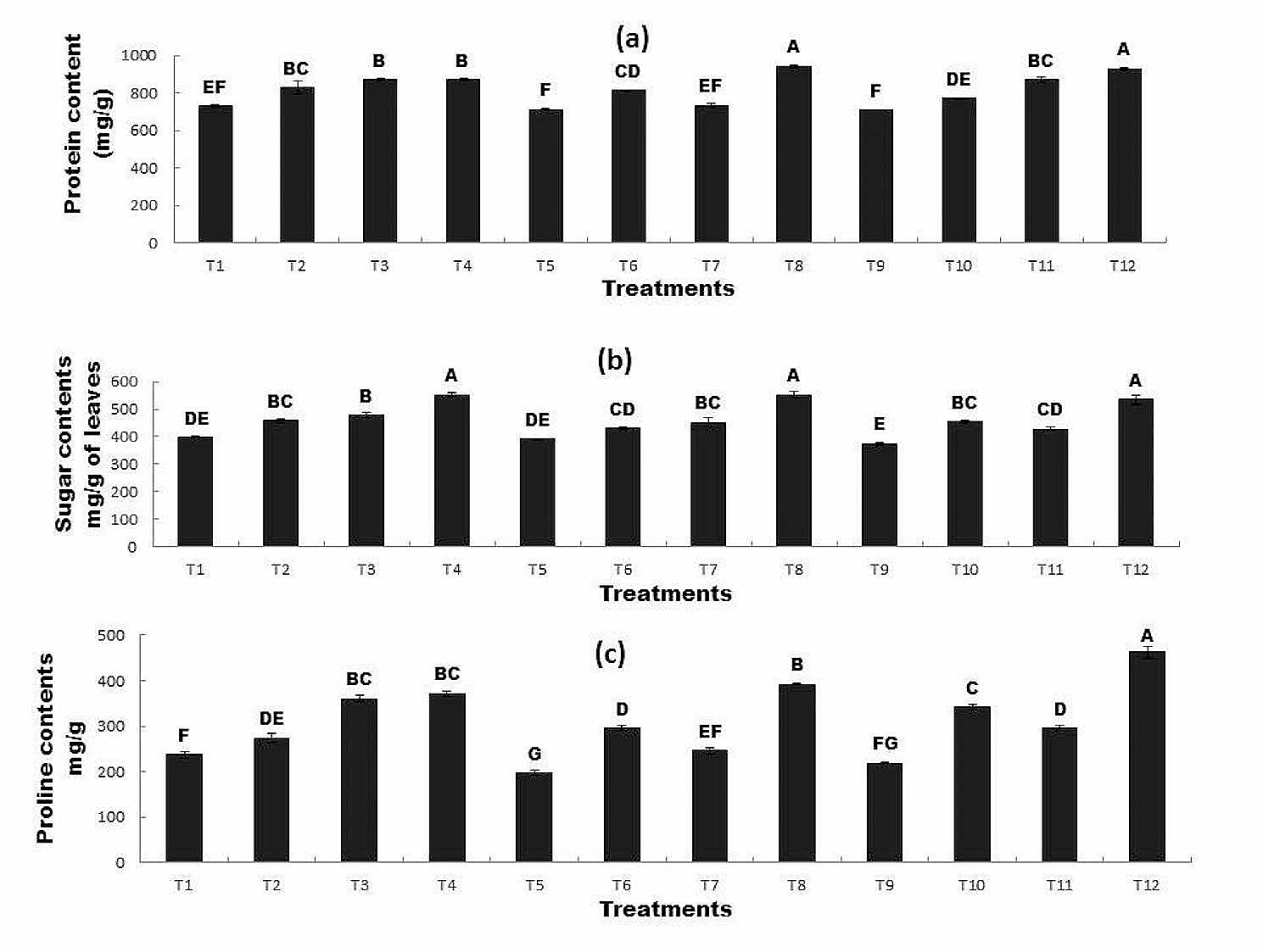
The Effect of R*P* and bacterial inoculation on (**a**) protein (**b**) sugar and (**c**) proline content of plant under Zn and Ni stress

The results showed that inoculation with *Bacillus mycoides* and application of R*P* significantly affected the protein content of wheat plants as compared to uninoculated seeds. Protein contents decreased in the presence of Ni and Zn (T5 and T9). However, the inoculation of seeds with *Bacillus mycoides* and application of R*P* significantly increased the protein content in T8 and T12. This increase was 29% and 27% higher than the control respectively (Fig. 4a). The sugar content of wheat also varied between inoculated and uninoculated seeds. The addition of Ni and Zn decreased sugar content (T5 and T9). However, the inoculation of seeds with *Bacillus mycoides* and the application of R*P* (T4, T8, and T12) significantly enhanced the sugar content as compared to all other treatments. The maximum sugar content was recorded in T8 (inoculated seed + Ni100mg/Kg + R*P* 0.2 g/Kg) followed by T4 (inoculated seed + R*P* 0.2 g/Kg) which was 39% and 38% higher than that of the control respectively (Fig. 4b). Inoculation with *Bacillus mycoides* and R*P* significantly affected the proline content of wheat plant seeds as compared to uninoculated seeds. Proline content decreased in the presence of Ni and Zn (T5 and T9). However, the combined application of R*P* and *Bacillus mycoides* significantly increased the proline content. The T12 (inoculated seed + Zn100mg/Kg + R*P*0.2 g/Kg) and T8 (inoculated seed + Ni 100 mg/Kg + R*P*0.2 g/Kg),showed 95% and 65% higher proline content as compared to control respectively (Fig. 4c). The Fig. 4 indicates that the T12 found effective in terms of protein, sugar and proline content followed by T8.

All treatments sharing common letter with similar bar pattern are similar otherwise vary significantly at *p* < 0.05.

T1 = control, T2 = seed + RR*P*0.2 g/Kg, T3 = inoculated seed, T4 = inoculated seed + R*P* 0.2 g/Kg, T5 = seed + 100 mg/Kg Ni, T6 = inoculated seed + 100 mg/Kg Ni, T7 = seed + Ni100mg/Kg + R*P*0.2 g/Kg, T8 = inoculated seed + 100 mg/Kg Ni + R*P*0.2 g/Kg, T9 = seed + 100 mg/Kg Zn, T10 = inoculated seed + 100 mg/Kg Zn, T11 = seed + 100 mg/Kg Zn + R*P*0.2 g/Kg, T12 = inoculated seed + 100 mg/Kg Zn + R*P*0.2 g/Kg.

### The Effect of R*P* and bacterial inoculation on (a) POD and (b) SOD contents under Zn and Ni stress

**Fig. 5:**
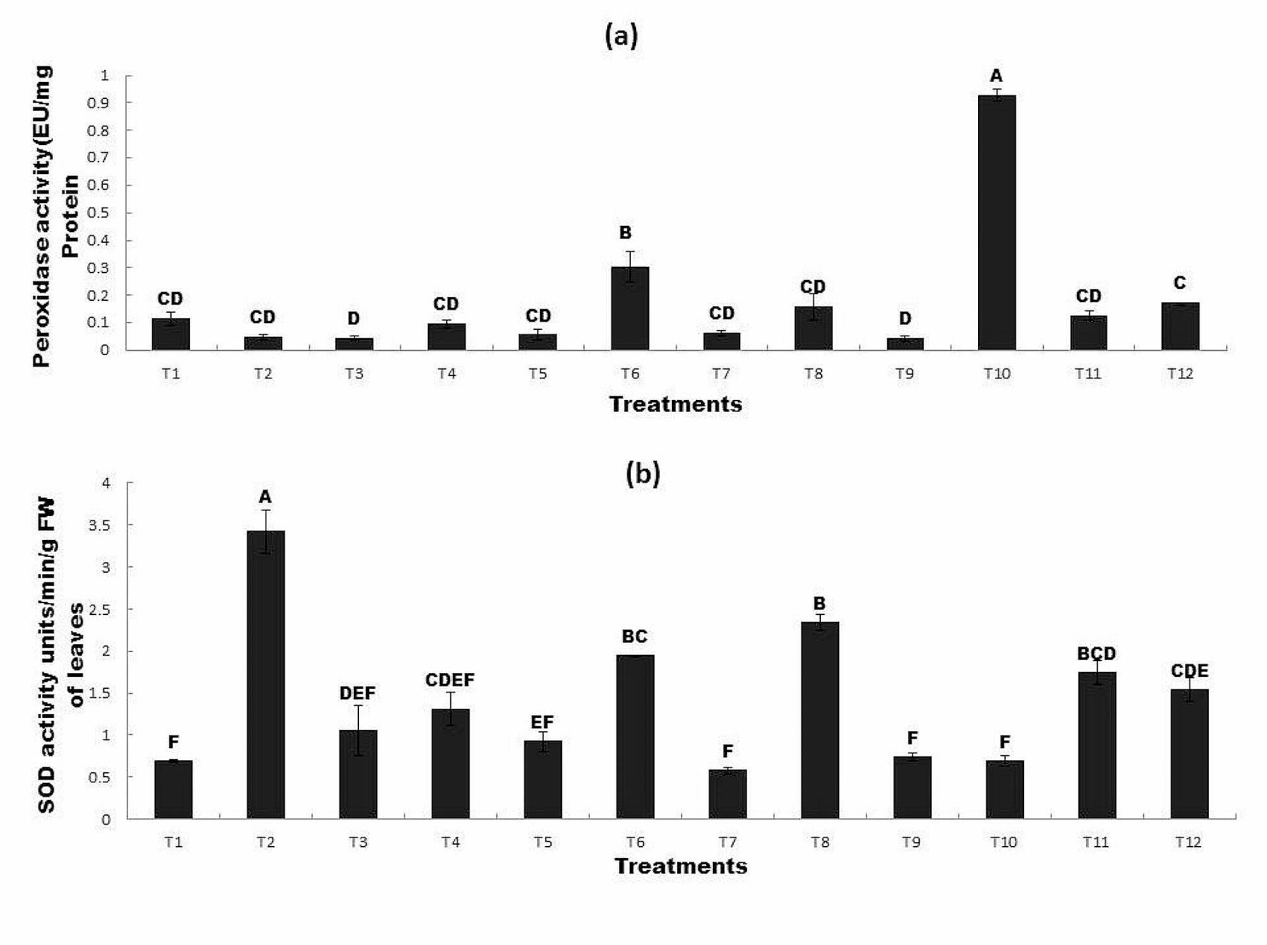
The Effect of R*P* and bacterial inoculation on (**a**) POD and (**b**) SOD contents under Zn and Ni stress

The peroxidase (POD) content of wheat plants varied between the inoculated and uninoculated seeds. The addition of Ni and Zn decreased POD content (T5, T9). However, inoculation with *Bacillus mycoides* significantly enhanced POD content as compared to all other treatments. The maximum POD content was observed in T10 (inoculated seed + Zn100mg/Kg) followed by T6 (inoculated seed + Ni100mg/Kg) which was approximately 710% and 166% higher than that of control respectively (Fig. 5a). The superoxide dismutase (SOD) content of wheat plants also varied between inoculated and uninoculated seeds. The addition of Ni and Zn decreased SOD content (T7, T9, and T10). The application of R*P* T2 (uninoculated seed + R*P*0.2 g/Kg) significantly enhanced the SOD content as compared to all other treatments. Similarly, the combined application of R*P* and inoculation with *Bacillus mycoides* significantly increased the SOD content (T8). The maximum SOD content was observed in T2 (uninoculated seed + R*P* 0.2 g/Kg) followed by T8 (inoculated seed + Ni100mg/Kg + R*P*0.2 g/Kg) which was approximately 395% and 238% higher than that of the control respectively (Fig. 5b). Figure 5 shows that POD content was maximum in T10 compared to all other treatments, whereas, SOD was higher in T2 than in all other treatments.

All treatments sharing common letter with similar bar pattern are similar otherwise vary significantly at *p* < 0.05.

T1 = control, T2 = seed + R*P*0.2 g/Kg, T3 = inoculated seed, T4 = inoculated seed + R*P* 0.2 g/Kg, T5 = seed + 100 mg/Kg Ni, T6 = inoculated seed + 100 mg/Kg Ni, T7 = seed + Ni100mg/Kg + R*P*0.2 g/Kg, T8 = inoculated seed + 100 mg/Kg Ni + R*P*0.2 g/Kg, T9 = seed + 100 mg/Kg Zn, T10 = inoculated seed + 100 mg/Kg Zn, T11 = seed + 100 mg/Kg Zn + R*P*0.2 g/Kg, T12 = inoculated seed + 100 mg/Kg Zn + R*P*0.2 g/Kg.

### The Effect of R*P* and bacterial inoculation on (a) plant Zn uptake and (b) plant Ni uptake under Zn and Ni stress

**Fig. 6:**
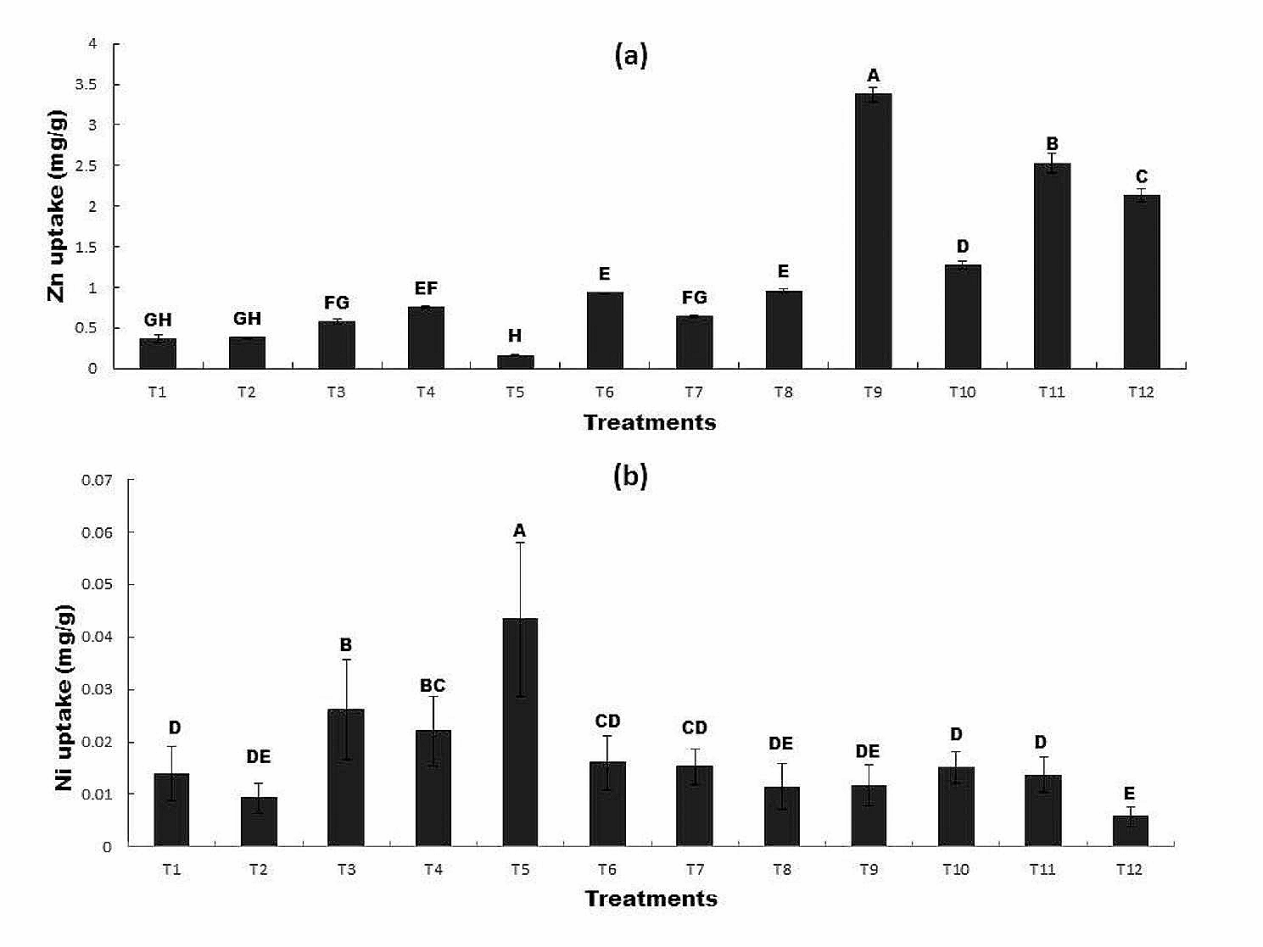
The Effect of R*P* and bacterial inoculation on (**a**) plant Zn uptake and (**b**) plant Ni uptake under Zn and Ni stress

The accumulation of Zn in wheat plants was significantly affected by the combined application of R*P* and *Bacillus mycoides* (T12). Zn uptake in all other treatments was not-significantly different from each other but in the presence of Ni, the inoculated seeds (T6) showed higher Zn uptake than control (T1) and (T5). The Zn accumulation was found maximum when Zn was added in the soil (T9), however the inoculation of wheat seeds (T10) reduced the Zn uptake as compared to T9 and 62% decrease was observed as compared to control. Similarly, Zn accumulation was significantly different between (T11) and (T12) (Fig. 6a). Ni accumulation was significantly affected by inoculation of *Bacillus mycoides* (T7) and the combined application of R*P* with *Bacillus mycoides* (T8). The plants showed a higher accumulation of Ni when Ni was added (T5) to the soil as compared to all other treatments. While inoculation of *Bacillus mycoides* (T6) reduced the Ni accumulation as compared to T5 and 63% decrease was observed. Similarly, the combined application of R*P* and inoculation of wheat seeds (T8) reduced Ni accumulation as compared to T5 **(**Fig. 6a & b).

All treatments sharing common letter with similar bar pattern are similar otherwise vary significantly at *p* < 0.05.

T1 = control, T2 = seed + R*P*0.2 g/Kg, T3 = inoculated seed, T4 = inoculated seed + R*P* 0.2 g/Kg, T5 = seed + 100 mg/Kg Ni, T6 = inoculated seed + 100 mg/Kg Ni, T7 = seed + Ni100mg/Kg + R*P*0.2 g/Kg, T8 = inoculated seed + 100 mg/Kg Ni + R*P*0.2 g/Kg, T9 = seed + 100 mg/Kg Zn, T10 = inoculated seed + 100 mg/Kg Zn, T11 = seed + 100 mg/Kg Zn + R*P*0.2 g/Kg, T12 = inoculated seed + 100 mg/Kg Zn + R*P*0.2 g/Kg.

### Accumulation of micro and macro nutrients by wheat plants

The results presented in Table 1 show the variation in Cu accumulation in wheat plants. The inoculation of *Bacillus mycoides* (T3) significantly affected the Cu content. The maximum Cu content was observed in T3 (inoculated seeds) which was 128% higher than that of the control and the minimum Cu content was observed in T5 (seed + Ni) which was 36% lower than that of the control. The inoculation of *Bacillus mycoides* and application of R*P* significantly affected the Mn content as compared to uninoculated seeds. The maximum Mn content was observed in T4 (inoculated seed + R*P*) which was 326% higher than that of the control. The combined application of inoculation with *Bacillus mycoides* and R*P* significantly affected the Na content as compared to all other treatments. The maximum Na^+^ content was observed in T4 (inoculated seed + R*P*) which were 449% of that in the control. Inoculation with *Bacillus mycoides* and the combined application of R*P* and inoculation of seeds with *Bacillus mycoides* significantly affected K content (T3, T4, and T12). The maximum K content was observed in T12 (inoculated seed + Zn + R*P*) followed by T4 (inoculated seed + R*P*) and T3 (inoculated seed) which were approximately 85%, 85%, and 84% higher than that of the control respectively. The combined application of inoculation with *Bacillus mycoides* and R*P* significantly affected the Fe content as compared to all other treatments. The maximum Fe content was observed in T12 (inoculated seed + Zn + R*P*) which was 216% as compared to the control and the minimum was observed in T5 (seed + Ni) which was 14% lower than that of control. Inoculation with *Bacillus mycoides* (T3) significantly affected the Ca content. The maximum Ca content was observed in T3 (inoculated seeds) which was 219% higher than that in the control. The combined application of inoculation with *Bacillus mycoides* and R*P* significantly affected the Mg content as compared to all other treatments. The maximum Mg content was observed in T8 which was 388% higher than that in the control.

**Table 1:** Accumulation of micro and macro nutrients by wheat plants

Treatments	Cu (mg/g)	Mn (mg/g)	Na (g/Kg)	K ( mg/g)	Fe (mg/g)	Ca (g/Kg)	Mg (g/Kg)
**T1**	1.9 ± 0.192 H	0.95 ± 0.020 F	0.73 ± 0.023G	1.05 ± 0.023 C	0.96 ± 0.011HI	0.77 ± 0.023 F	0.51 ± 0.075E
**T2**	3.02 ± 0.061EF	2.47 ± 0.133BCDE	2 ± 0.043E	1.42 ± 0.053B	1.37 ± 0.025G	1.68 ± 0.086CD	0.76 ± 0.033CDE
**T3**	4.8 ± 0.214 A	3.55 ± 0.127AB	3.47 ± 0.074B	1.95 ± 0.017 A	1.91 ± 0.037E	2.47 ± 0.106 A	0.85 ± 0.214CDE
**T4**	4.33 ± 0.015AB	4.04 ± 0.287 A	4.04 ± 0.049 A	1.96 ± 0.055 A	2.22 ± 0.035D	1.84 ± 0.049BCD	1.03 ± 0.018 C
**T5**	1.21 ± 0.069I	2.11 ± 0.775CDEF	1.07 ± 0.073G	0.18 ± 0.010 F	0.82 ± 0.013I	0.92 ± 0.075 F	0.61 ± 0.023DE
**T6**	2.2 ± 0.028GH	1.81 ± 0.331DEF	1.62 ± 0.074 F	0.316 ± 0.029 F	1.14 ± 0.021 H	2.22 ± 0.028AB	1.76 ± 0.040B
**T7**	2.40 ± 0.116GH	2.37 ± 0.020BCDE	2.09 ± 0.033DE	0.84 ± 0.055D	1.70 ± 0.057 F	1.60 ± 0.015DE	1.60 ± 0.025B
**T8**	3.71 ± 0.808CD	3.16 ± 0.018ABC	2.44 ± 0.097 C	1.15 ± 0.025 C	2.28 ± 0.033D	1.75 ± 0.051CD	2.52 ± 0.054 A
**T9**	1.2 ± 0.033I	1.18 ± 0.021EF	1.77 ± 0.035EF	0.61 ± 0.013E	1.34 ± 0.058G	1.18 ± 0.024EF	0.91 ± 0.020CD
**T10**	3.27 ± 0.050DE	2.88 ± 0.033ABCD	2.54 ± 0.104 C	0.84 ± 0.018D	2.51 ± 0.038 C	1.66 ± 0.032D	1.56 ± 0.036B
**T11**	2.52 ± 0.123FG	1.99 ± 0.071CDEF	2.38 ± 0.045CD	0.74 ± 0.014DE	2.78 ± 0.035B	0.96 ± 0.239 F	1.13 ± 0.057 C
**T12**	4.23 ± 0.045BC	3.70 ± 0.075AB	3.47 ± 0.100B	1.96 ± 0.039 A	3.05 ± 0.025 A	2.11 ± 0.032ABC	1.86 ± 0.035B

All treatments sharing common letter with similar bar pattern are similar otherwise vary significantly at *p* < 0.05.

T1 = control, T2 = seed + R*P*0.2 g/Kg, T3 = inoculated seed, T4 = inoculated seed + R*P* 0.2 g/Kg, T5 = seed + 100 mg/Kg Ni, T6 = inoculated seed + 100 mg/Kg Ni, T7 = seed + Ni100mg/Kg + R*P* 0.2 g/Kg, T8 = inoculated seed + 100 mg/Kg Ni + R*P* 0.2 g/Kg, T9 = seed + 100 mg/Kg Zn, T10 = inoculated seed + 100 mg/Kg Zn, T11 = seed + 100 mg/Kg Zn + R*P* 0.2 g/Kg, T12 = inoculated seed + 100 mg/Kg Zn + R*P* 0.2 g/Kg.

## Discussion

Zinc is a crucial nutrient that promotes plant growth and development [40]. Zinc (Zn) acts as a cofactor for various enzymes and regulates enzyme activity [41] however, Zn toxicity has a negative effect on vegetation [42]. Likewise, Ni is an important micronutrient that helps plant growth and development, and plays an important role in various biological functions [43]. Zn is a vital component of numerous enzymes such as glyoxalase-I and urease which are required for nitrogen metabolism in plants, especially in higher plants [44]. Several studies reported that Ni toxicity significantly decreases nutrient uptake in plants [43] and causes nutrient imbalance [45].

In the present study, the data suggest that the accumulation of heavy metals (Zn and Ni) in the soil had toxic effects on plant growth which significantly inhibited plant height, shoot length, leaf length, number of leaves, root length, fresh weight enzyme activity, and nutrient content. In this study, the plant height, growth, and biomass, when inoculated with *Bacillus mycoides* in combination with R*P* significantly affected wheat plants which is in close agreement with the results of Siddique, et al. [46], who demonstrated that the damage to soil, that is soil pH, presence of various elements and addition of toxic metals can directly or indirectly lead to reduced plant growth and negatively disrupt the physiological and molecular activities of plants. de Oliveira Mendes, et al. [47] found that inoculation of seeds with plant-promoting bacteria significantly enhanced the plant height, soil diameter, and plant biomass, which could be due to the manufacturing of organic acids (e.g., gluconic, formic, and citric acids) by these strains. Our results indicated that higher concentrations of Zn and Ni inhibited seed germination. Previous studies have also documented a detrimental effect of Zn on plant growth parameters, which is in agreement with the findings of Saleem et al. [48], who demonstrated that cadmium stress can cause chlorosis and variability in the lipid membrane thus preventing plant growth. Similar reports were found in the present study in which the application of R*P* improved the germination percentage in the presence of Zn. R*P* has a positive effect on Zn toxicity during seed germination.

Rizvi, et al. [36] also noted that R*P* contributes to many important mechanisms of plant growth in cadmium-contaminated soil by decreasing cadmium (Cd^+ 2^) transfer from soil to plant. The Zn toxicity decreased the root length and shoot length as well as the area of leaves in tomato plants (*Solanum lycopersicum* L.) [49]. These results also supported by the findings of Sharma et al., [50], who reported that the noxious influence of Cr on plant growth and development resulted in the alteration of the germination process in plants. Therefore, total plant matter production and yield of plants are affected by exposure to high levels of chromium. Similarly, during the present investigation, the presence of Zn and Ni in soil suppressed the length of roots and shoots as well as the number and width of leaves but inoculation of *Bacillus mycoides*, application of R*P* and combine the application of R*P* and inoculation of *Bacillus mycoides* significantly reduced the negative effect of Ni and Zn which resulted in increased length of roots and shoots as well as number and width of leaves. Tahir et al. [51] also reported similar findings that combined application of plant growth-promoting bacteria with phosphate fertilizer increased the grain production of wheat plants which was higher than the un-inoculated phosphate fertilizer. Similarly, Khan et al. [52] also supported the current findings that phytohormone production through plant growth promoting rhizobacteria contributes to plant growth and crop yield. *Bacillus pumilus* increases plant fresh mass by producing hormones such as indole acetic acid. Rahman, et al. [53] also suggested that hormone production increases the length of plant roots and shoots, as well as leaf size, which enhances the fresh weight of the corn plant. Many species of *Bacilli* are also responsible for the bioavailability of macro and micronutrients in the soil, which has a beneficial effect on the fresh weight of plants [54] This is in agreement with the present findings in which Zn and Ni toxicity affected the fresh weight of wheat, but seeds inoculated with *Bacillus mycoides* decreased the effect of heavy metals and enhanced plant fresh weight.

Higher concentrations of copper can cause a decline in leaf protein content [55]. Khan, et al. [52] noted that wheat plants treated with different concentrations of lead (Pb), cadmium (Cd), and zinc (Zn), can lead to stressed plant growth which resulted in protein content reduction and physiological imbalance. Similar results are reported in the present investigation in which protein content was decreased in the wheat plants when the wheat plants are grown over Zn and Ni contaminated soil (T5, T9). Moreover, inoculation with *Bacillus mycoides* and application of R*P* enhanced the protein content. The present study showed similar findings to those of Nosheen et al. [56] who demonstrated that the application of plant growth-promoting rhizobacteria impacts the crude protein of safflower seeds and then increases the protein content under stress conditions.

Shen et al. [57] demonstrated that the content of leaves soluble sugar of *K. obovata* seedlings decreased with the increase of a copper (Cu) concentration. Kumaret al. [55] also suggested that reduction in total sugar content in wheat seedlings under higher concentrations of copper (Cu) which supported our findings in which the lower concentration of Ni and Zn had an adverse influence on total sugar content in the wheat plant and the total sugar content was reduced due to the toxicity of Zn and Ni. However, when wheat plant seeds were inoculated with *Bacillus mycoides* and R*P* was applied, the sugar content in wheat plant leaves increased, which is consistent with the findings of Kumar et al. [58], who suggested that the sugar content of leaves increased significantly with grafting against *Rhizobium* and fertilizers. The highest value of leaf sugar content was noted down in plants by inoculating bacteria with treated fertilizers in plants, in the same way as Zincircioğlu and Yalçin [59] who determined that increased sugar content in sugar beets by inoculating nitrogen-fixing bacteria and phosphate-soluble bacteria together.

The result of this study showed that proline content in wheat plant leaves was decreased when Zn and Ni were added to the soil and comparable results were reported by Qiaoet al. [21] who suggested that the addition of Zn and cadmium (Cd) in soil affected the plant growth by lowering proline content. Moreover, in the current study, inoculation with *Bacillus mycoides* and application of R*P* enhanced proline content in wheat plant leaves. Similarly, Kalayu, et al. [60] reported that inoculation of phosphate solubilizing microorganisms in plants enhanced solubilization and increased enzyme activity (e.g. proline). The increase in proline content may be due to the excretion of organic acids by microbes in the soil, which helps the plant to overcome metal stress. The higher amount of organic acids may be responsible for the reduction in the pH of the soil and is helpful in increasing the activity of soil enzymes [61].

Important antioxidant enzymes are superoxide dismutase (SOD) and peroxidase, which deceptively contribute to reactive oxygen species (ROS) and are associated with plant growth through the instruction of reactive oxygen species generation and alteration. Liu et al. [62], Li et al. [63] reported that higher concentrations of lead (Pb); inhibit antioxidant enzymes in plants. Peroxidase (POD) can convert H_2_O_2_ to H_2_O and Superoxide dismutase (SOD) which breakdown of O_2_ - radicals to H_2_O_2_ and O_2_. Sytar et al. [64] also confirmed that plants induced oxidative stress by heavy metals resulting in the formation of superoxide radicals, which are collectively termed reactive oxygen species such as singlet oxygen (O_2_), hydroxyl radical (HO), and hydrogen peroxide (H_2_O_2_). In the present study, zinc (Zn) and nickel (Ni) induced Superoxide dismutase and peroxidase contents in the presence of zinc (Zn) and nickel (Ni). Nevertheless, inoculation with *Bacillus mycoides* and the application of R*P* protected wheat plants from zinc (Zn) and nickel (Ni) stress by inducing SOD and POD. Bakhtiyarifar, et al. [65] demonstrated the ability of many bacterial species including *Serum Bacillus* and *Enterobacteria* to solubilize phosphate which can help to support plant growth and resistance against heavy metals in plants.

Under higher Cr concentration Cr plant nutrient uptake is affected, leading to nutrient shortages in numerous agrarian crop plants [66]. Nutrients were significantly decreased in tomato plants treated with 50 and 100 mg/L Cr [67]. Similarly, Herath et al. [67] reported adverse effects of Cr on iron absorption in *Lycopersicum esculentum*. Rai et al. [68] demonstrated that the inhibition of nutrient transport under the toxic heavy metals in soil is due to plasma reluctance to H + ATPs action. This evidence supports our findings that Ni and Zn have adverse effects on nutrient uptake in wheat plants, resulting in nutrient deficiencies in wheat plants. Moreover, in the current study, *Bacillus mycoides* and R*P* increased the absorption of nutrients as compared to uninoculated plants consistent with the findings of Verma et al. [69], who reported that *Bacillus* species have been shown to improve nutrient availability in plants. Kidd, et al. [70] verified that bacteria improve the selective absorption of essential nutrients Ca, Mg, K, and Na. Bacteria can transport, mineralize, and mobilize soil P, K, Fe, and N. Jamil et al. [71] also supported the presented results in which they demonstrated that consortium of some *Bacillus spp*. rises the phosphorous and calcium content which reduces the uptake of nickel in plants in nickel-contaminated soil. These decreases in nutrient availability can inhibit the growth, development, and productivity of crops in heavy metal-contaminated soils [72].

## Conclusions

It is concluded from the present investigation that the presence of heavy metals i.e. Zn and Ni adversely affected the growth of the wheat (*Triticum aestivum*) plant but the severity of nickel (Ni) towards growth inhibition was prominent as compared to Zinc (Zn) however, the isolated endophytic bacteria (*Bacillus mycoides )* found beneficial alone (T11) and in combination with and RP (T12) which suppressed the toxicity of Zn and Ni and showed significant results in term of wheat (*Triticum aestivum*) shoot length, leaf width, protein contents, and sugar contents. Combined treatment with *Bacillus mycoides* and RP (T12) was found to be effective for significant growth promotion of plant agronomic, biochemical, and antioxidant responses. Moreover, the accumulation of Ni and Zn in wheat plants was also inhibited in T12. From the above findings it is concluded that *Bacillus mycoides* activity was enhanced by R*P* which promoted plant growth under Ni and Zn contamination. In the future, the combination of (*Bacillus mycoides* with R*P* could be an effective remedy to enhance plant growth under heavy metal stress conditions.

## Methods

### Sample collection

The roots of *Viburnum grandiflorum* were collected from District Poonch (Davi Gali) at an elevation of 5000 ft. Healthy and mature plants were selected for the bacterial isolation. The roots of the selected plant *Viburnum grandiflorum* were carefully cut and packed in zip–lock plastic bags. The samples were then transported to the laboratory for further research.

### Isolation of endophytes bacteria

Small pieces of root were made with a sterilized knife. The roots were washed with sterilized water 5–6 times for 2–5 min. Sterile root pieces were macerated in phosphate buffer (PB) at pH 7.0. After maceration the samples were ground with a disinfected pestle and mortar in 9.5 ml of final buffer wash of distilled water and ground. Ten milliliters of the ground sample, placed in a Falcon tube. Two replicates of each sample were prepared and centrifuged at 3000 rpm for 15 min. Afloat was collected and serial dilutions of this extract were prepared in phosphate buffer (10^− 5^, 10^− 6^, and 10^− 7^). From each dilution, a 0.1 ml extract was inoculated on separate Petri plates containing nutrient agar media. The inoculated petri plates were incubated at 37 C° bacterial growth was observed after 48–72 h. Bacterial isolates were selected from the plates and purified using streaking. The isolation process was repeated until pure colonies were obtained. Different colonies were obtained based on their morphology and color. Gram staining was performed to identify gram-positive and negative bacteria [73].

### Identification of endophytic bacteria

Genomic DNA was extracted from the bacterial strains using a Bacterial Genomic DNA Kit [74]. The bacterial strains were grown in nutrient broth and kept overnight for the extraction of genomic DNA as reported by Naeem et al. [75] and DNA was stored at -20^o^C.

### 16S rRNA sequencing of endophytic bacteria

Amplified and pure PCR products of approximately 1400 bp were sequenced using two primers, as described below. (BioSystems, USA). Sequencing products were resolved on an Applied Biosystems 3730XL automated DNA sequencing system (Applied BioSystems, USA) at Macrogen, Inc., Seoul, Korea. The following primers were used.

- 27F AgA gTT TgA TCM TGG CTC Ag (amplification).
- 1492R TAC ggY TAC CTT gTT ACg ACT T (amplification).
- 518F CCA gCA gCC gCg gTA ATA Cg (Sequencing).
- 800R TAC CAg ggT ATC TAA TCC (Sequencing).

### Phylogenetic tree construction

For phylogenetic study of isolated bacterial strains with other strains, MEGA 6.06 (http://megasoftware.net/) software was used to construct a dendogram with Neighbor-Joining method and bootstrap test replicated 1000 times [76].

### Preparation of inoculum

Nutrient broth medium was sterilized at 121 °C for 20 min. Sterile media (250 ml sterile media were added to the flask and 24 h old bacterial culture was inoculated into the flask and incubated in a shaker incubator (NB-205LF) at 37 °C and 150 × g, and the bacterial count was monitored until sufficient bacterial count was reached.

### Seed inoculation

Wheat (*Triticum aestivum L*.) cultivar viz. Faisalabad-2008 (Faisalabad-2008) is a very famous wheat variety used in Pakistan. This variety is known for its high grain yield and drought tolerance and was obtained from the National Agricultural Research Center (NARC) in Islamabad. The surface of the seeds was cleaned by washing with 95% ethanol and then immersing in 10% Clorox for 2 to 3 min after that the seeds were washed successively to 2–3 times in sterile water (H_2_O). After sterilization the seeds were dipped in the prepared bacterial inoculum with an optical density of (O.D) 1 at 660 nm for 3–4 h. and sown in potted sterilized soil.

### Preparation of Zinc and nickel solution

Ni and Zn solutions were prepared in sterile water by dissolving 100 mg zinc sulfate in 100 ml of water. Similarly, 100 mg Ni was dissolved in 100 ml of water. Both Znand Ni solution were added and mixed well in 1 kg of soil before seed sowing (100 mg/100 ml/kg) [77].

### Addition of RP

Rock phosphate (RP) was collected from Hazara (34° 16.834 N and 73° 18.911E), Khyber Pakhtunkhwa, Pakistan. R*P* powder was directly added to the soil after seed sowing. The total amount of R*P* that was added to the soil was 0.2 g/kg soil in each and every pot. Calculations were performed as described by Bell et al. [78]

Calculations:

1 $$\begin{array}{l} \text{R}\text{P} =100\text{g}/\text{h}\text{a}\text{c}\\\text{R}\text{P} =\text{100,000}\text{g}/\text{2000,000}\text{k}\text{g}\,\text{s}\text{o}\text{i}\text{l}\\\text{R}\text{P} =0.05\text{g}/\text{k}\text{g}\,\text{s}\text{o}\text{i}\\\text{A}\text{s}\,\text{R}\text{P}\,\text{h}\text{a}\text{s}\,46\text{\%}\,\text{s}\text{o},\,100/46=2.17\\\text{R}\text{e}\text{q}\text{u}\text{i}\text{r}\text{e}\text{d}\,\text{R}\text{P}\,=0.05\text{*}2.17 =0.1085\text{g}/\text{k}\text{g}\,\text{s}\text{o}\text{i}\text{l}\\\text{A}\text{b}\text{o}\text{u}\text{t}\,0.2\text{g}\,\text{R}\text{P}\,\text{w}\text{a}\text{s}\,\text{a}\text{d}\text{d}\text{e}\text{d}/1\text{K}\text{g}/\text{p}\text{o}\text{t}.\end{array}$$

### Treatments applications

Inoculated and uninoculated seeds of wheat plants were added to the soil. The RP and solutions of Zn and Ni were also added to the soil. Twelve treatments with three replicates were prepared and ten seeds of wheat were sowed in each. The table of treatments is given below. Plants were harvested after 21 d of seeding for further study (Table 2).

**Table 2:** Treatment application

Treatments	Conditions
T1	Control
T2	Seed + R*P* 0.2 g/kg
T3	Seed + *Bacillus mycoides*
T4	Seed + *Bacillus mycoides* + R*P* 0.2 g/Kg
T5	Seed + Ni 100 mg/Kg
T6	Seed + Ni 100 mg/Kg + *Bacillus mycoides*
T7 T8 T9 T10 T11 T12	Seed + Ni 100 mg/Kg + R*P* 0.2 g/Kg Seed + Ni 100 mg/Kg + R*P* 0.2 g/Kg + *Bacillus mycoides* Seed + Zn 100 mg/Kg Seed + Zn 100 mg/Kg + *Bacillus mycoides* Seed + Zn 100 mg/Kg + R*P* 0.2 g/Kg Seed + Zn 100 mg/Kg + R*P* 0.2 g/Kg + *Bacillus mycoides*

### Plants nutrient analysis

A perchloric-acid digestion scheme was used to determine the contents of various nutrients in wheat plants [79]. The aqueous solution of nitric acid, sulfuric acid and perchloric acid at 5:1:0.1 (6.5 mL) was added to 0.25 g of plant leaf sample in a 50 mL flask. The mixture was boiled on a hot plate in the fume hood to complete the digestion. The appearance of white fumes from the mixture in the flask indicated the completion of the plant digestion process. Thereafter, a few drops of distilled water were added to the flask and the mixture was allowed to cool down. Completely digested plant extract samples were transferred to volumetric flasks (50 mL) and the mixture volume was increased to 50 mL by the addition of distilled water. The digested plant extract samples were filtered through Whatman No.42 filter paper and stored for elemental analysis. The concentrations of different elements and nutrients present in the plant samples were measured using a Shimadzu AA-670 Atomic Absorption Spectrophotometer.

2 $$\begin{array}{l} \text{C}\text{a}\text{t}\text{i}\text{o}\text{n}\text{s}\,\text{i}\text{n}\,\text{p}\text{l}\text{a}\text{n}\text{t}\text{s}= \left(\text{p}\text{p}\text{m}\,\text{i}\text{n}\,\text{e}\text{x}\text{t}\text{r}\text{a}\text{c}\text{t} -\text{b}\text{l}\text{a}\text{n}\text{k}\right)\times \frac{\text{A}}{\text{W}}\times \text{d}\text{i}\text{l}\text{u}\text{t}\text{i}\text{o}\text{n}\,\text{f}\text{a}\text{c}\text{t}\text{o}\text{r}\\\text{A}=\text{T}\text{o}\text{t}\text{a}\text{l}\,\text{v}\text{o}\text{l}\text{u}\text{m}\text{e}\,\text{o}\text{f}\,\text{e}\text{x}\text{t}\text{r}\text{a}\text{c}\text{t}\,\left(\text{m}\text{L}\right)\\\text{W}=\text{W}\text{e}\text{i}\text{g}\text{h}\text{t}\,\text{o}\text{f}\,\text{d}\text{r}\text{y}\,\text{p}\text{l}\text{a}\text{n}\text{t}\end{array}$$

### Seeds germination percentage

The germination percentage was recorded on the fifth day of sowing and wheat plants were uprooted after 21 days of sowing for various analyses.

### The estimation of plant biochemical

The protein content of fresh wheat plant leaves was examined using the method described by Dahot [80]. About 0.1 g of wheat plant leaves was grinded in phosphate buffer. The mixture was centrifuged at 3000 rpm for 10 min. The supernatant was mixed with purified water and the volume was increased to 1 ml. The resulting solution was added to 1 ml of alkaline CuSO4 reagent and shaken for 10 min following which folline reagent was added to the solution and incubated for 30 min at 28 ± 2ºC. Absorbance was measured at 650 nm using a spectrophotometer. Wheat proline was extracted from fresh leaves by using the method of Bates, et al. [80] and Ashraf [81] using the formula given.

3 $$\text{P}\text{r}\text{o}\text{l}\text{i}\text{n}\text{e} =\text{K}\text{*}\text{D}\text{i}\text{l}\text{u}\text{t}\text{i}\text{o}\text{n}\,\text{F}\text{a}\text{c}\text{t}\text{o}\text{r}\text{*}\text{O}\text{p}\text{t}\text{i}\text{c}\text{a}\text{l}\,\text{d}\text{e}\text{n}\text{s}\text{i}\text{t}\text{y}/\text{w}\text{e}\text{i}\text{g}\text{h}\text{t}\,\text{o}\text{f}\,\text{t}\text{h}\text{e}\,\text{s}\text{a}\text{m}\text{p}\text{l}\text{e}.$$

About 0.1 g of fresh wheat plant leaves were used to examine proline content. Wheat plants leaves (0.1 g) were placed in 10 ml of methanol. The mixture was centrifuged at 3000 rpm for 10 min. After 24 h the mixture was placed in refrigerator. After 24 h, the mixture was again centrifuged.1 ml and 5% phenol was added and incubated at room temperature for at least 1 h. Then, sulfuric acid (2.5 ml of sulfuric was added and the absorbance was measured at 490 nm. The Sugar content of the wheat leaves was estimated following the method described by Gill et al., [82]. Fresh wheat (0.5 g) was ground in 10 ml of distilled water. The extract was centrifuged at 3000 rpm for 5 min. The supernatant was collected, mixed with 1 mL of 80% phenol and incubated at room temperature for 1 h. After incubation, 5 ml of sulfuric acid was added. The samples were again incubated for 4 h at room temperature. The absorbance was measured at 420 nm.

The suger content was determined using the formula.

4 $$\eqalign{& Total{\text{ }}sugar{\text{ }}\left( {\mu g/g} \right) = \cr& \frac{{k{\text{ }}value{\text{ }}of{\text{ }}sugar*Dilution{\text{ }}factor*absorbance{\text{ }}value\left( {mg} \right)}}{{Weight{\text{ }}of{\text{ }}sample{\text{ }}\left( g \right)}} \cr}$$

### The estimation of plant antioxidant enzymes

The peroxidase activity of wheat plant leaves was examined using the method described by Van Assche et al. [83]. Approximately 1 g of cold wheat leaves were used to examine the POD antioxidant enzyme.1 g of frozen wheat leaves was placed in an icy mortar with 0.5 M of calcium chloride solution. The extract was centrifuged at 1000 rpm for 10 m and the supernatant was transferred to clean test tubes and stored in a freezer. The pellet of the cell wall extract remaining in the centrifuge tube was 2.5 mL in ice cold mixed calcium chloride solution 0.5 M and centrifuged again. The Superoxide activity of wheat plant tissues was assessed using the techniques described by Naeem et al. [84]. About 0.2 g of frozen wheat leaves were used to examine the SOD enzyme, which was placed in 1 g polyvinylpyrrolidone (PVP polyvinylpyrolidone) + 0.0278gNaEDTA solutions in a cooled pestle and mortar, and the mixture was centrifuged at 4 °C for 10 min. The supernatant was collected and adjusted to 8 ml by adding phosphate buffer at pH 7. The mixture further processed and mixed with 0.0278 g NaEDTA + 1.5 g Methionine + 0.04 g Nitro blue tetrazolium chloride (NBT) in 100 ml of phosphate buffer (pH 7.8). Three types of assays were performed,1st Reference, 2nd Blank and 3rd Reaction mixture.

The reference sample was mixed well and kept in the dark, and the reaction mixture was kept in the light chamber for approximately 20 min. Absorbance was measured at 560 nm using a spectrophotometer.

### Statistical analysis

The experiment was conducted using a completely randomized design (CRD). The data were analyzed, and values were differentiated by comparing means using analysis of variance (annova) in Statix 8.1. Three replicates of each treatment were made [85].

### Electronic supplementary material

Below is the link to the electronic supplementary material.

Supplementary Material 1

## Data Availability

The datasets used and/or analyzed during the current study will be available from the corresponding author upon reasonable request. The bacterial 16SrRNA DNA sequence was constructed. “The datasets generated and/or analyzed during the current study are available in the name of [Shahzad,A., Ferdose,S. and Hameed,S.] in the GeneBank repository, with a Web link as given: https://www.ncbi.nlm.nih.gov/nucleotide/MW979613.1?report=genbank&log$=nucltop&blast_rank=1&RID=BHYJ6SFW013 with an accession number (Acc MW979613) to datasets]
