# Supplementary material for: Combined effect of endophytic Bacillus mycoides and rock phosphate on the amelioration of heavy metal stress in wheat plants
Source: BMC Plant Biol. 2024 Feb 20;24:125. doi: 10.1186/s12870-024-04812-3 (PMC10877812; doi:10.1186/s12870-024-04812-3)
Supplement: Supplementary file 1 — Supplementary Material 1 [file 12870_2024_4812_MOESM1_ESM.docx]

**Table S1 Endophytic Bacterial characterization Isolated from *Viburnum grandiflorum roots***

|  | **Plant/colony** | **Color** | **Size** | **Margin** | **Texture** | **Gram staining** | **Types of bacteria** | **IAA** | **PSB** | **HCN** |  |
| --- | --- | --- | --- | --- | --- | --- | --- | --- | --- | --- | --- |
|  | *Vibernum grandiflorum*  (a)colony *Bacillus mycoides* MIU (Acc MW979613) | White | Small | irregular | Flate | +ve | *Bacilli* | *+* | *+* | *+* | |
|  | *Vibernum grandiflorum*  (b)colony  *Vibernum grandiflorum*  (c)colony  *Vibernum grandiflorum*  (d)colony  *Agrococcus terreus (*MW 979614) | White  Yellow  Yellow | Large  Small  Large | Irregular  Smooth  Smooth | Flate  Flate  Flate | +ve  +ve  +ve | *Bacilli*  *Bacilli*  *Bacilli* | *-*  *-*  *+* | *+*  *-*  *+* | *-*  *-*  *+* |  |
|  |  |  |  |  |  |  |  |  |  |  |  |

**Soil physiochemical characteristics**

Soil physicochemical characteristics such as texture, pH, organic matter were determined using the method as described by Jackson (1964).

**Soil physicochemical characteristics**

Soil physicochemical characteristics are present in Table 1. The pH of the soil is 6.5 and The organic matter was 0.874% in the soil . The total Phosphorus was 0.71 and Nitrate-Nitrogen was 2.11 with EC 51.4

**Table S2.** **Soil physicochemical properties**

| **physicochemical properties** | |
| --- | --- |
| pH | 6.5 |
| EC (dS cm^-1^) | 51.4 |
| Color (Hue-value/chroma) | 7.5YR-7/6-Reddish yellow |
| Nitrate-Nitrogen (mg/kg) | 2.11 |
| Total Phosphorus (mg/kg) | 0.71 |
| Organic matter (%) | 0.774 |


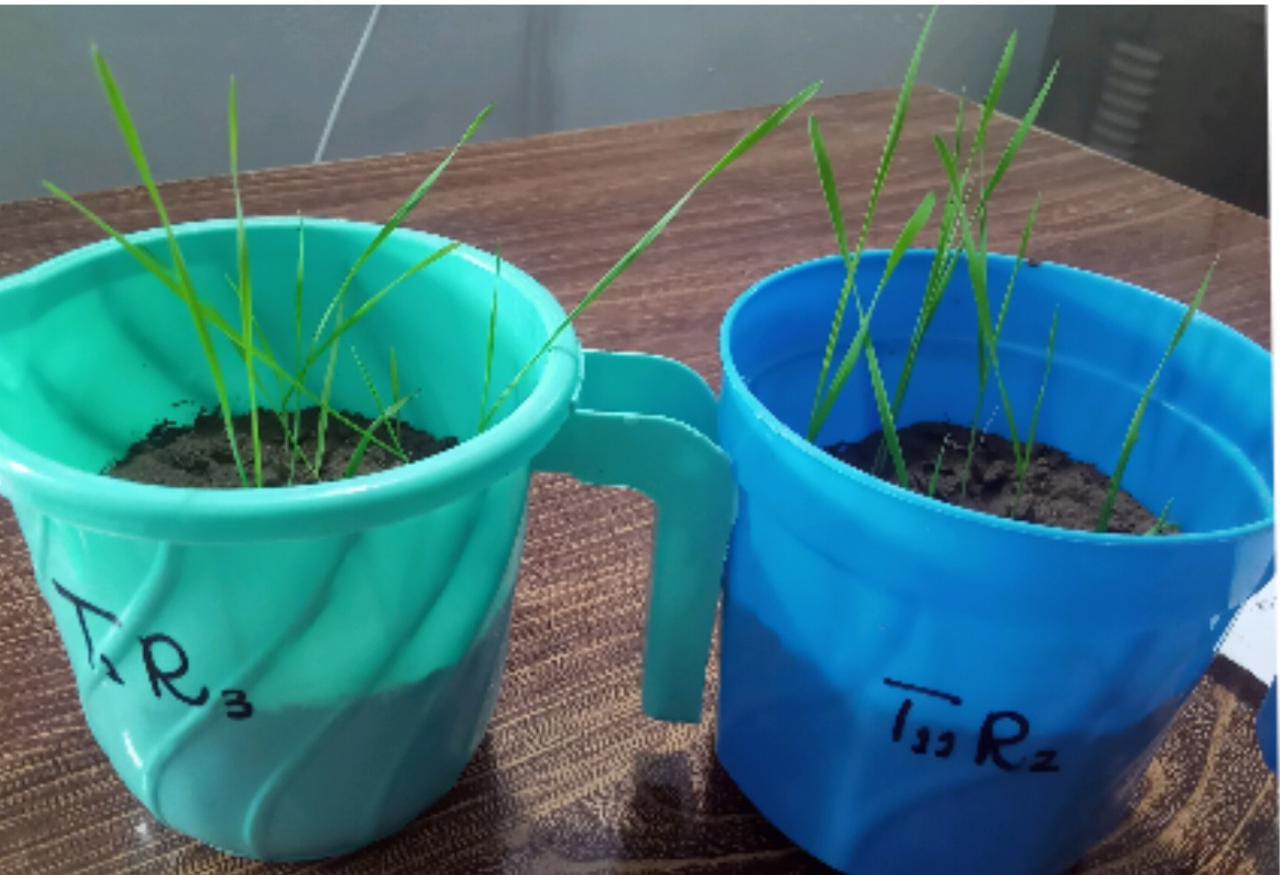

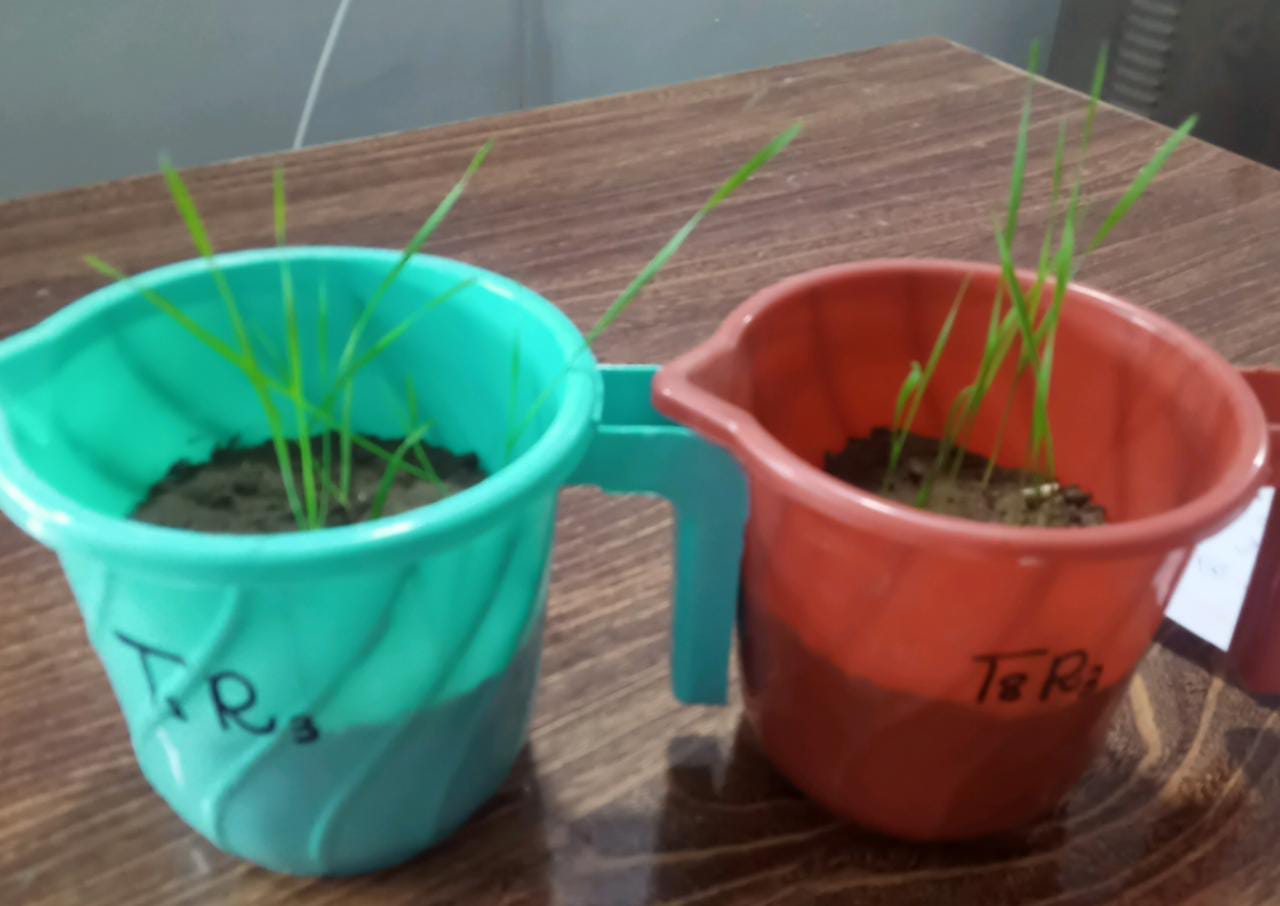

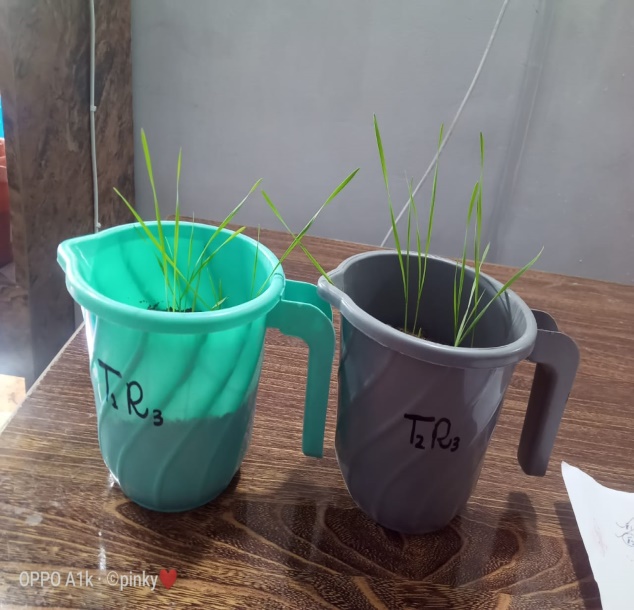

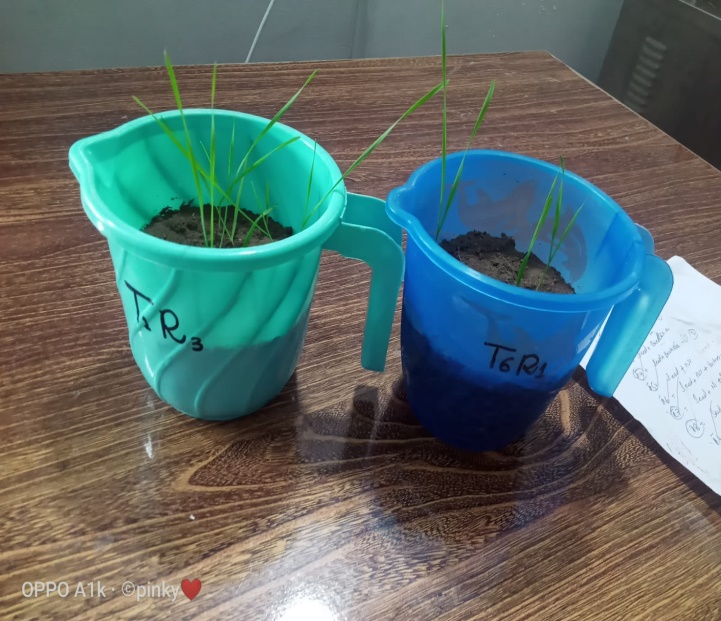


**Figure S1 . Pot experiment showing plant growth in different treatment applications**
